# Supplementary material for: Causal Relationship between Aging and Anorexia Nervosa: A White-Matter-Microstructure-Mediated Mendelian Randomization Analysis
Source: Biomedicines. 2024 Aug 16;12(8):1874. doi: 10.3390/biomedicines12081874 (PMC11351342; doi:10.3390/biomedicines12081874)
Supplement: Supplementary file 1 [file biomedicines-12-01874-s001.zip › Supplementary Figure/Figure S4/Singe SNP plot of MR analysis for TL to ubm-b-1951.pdf]

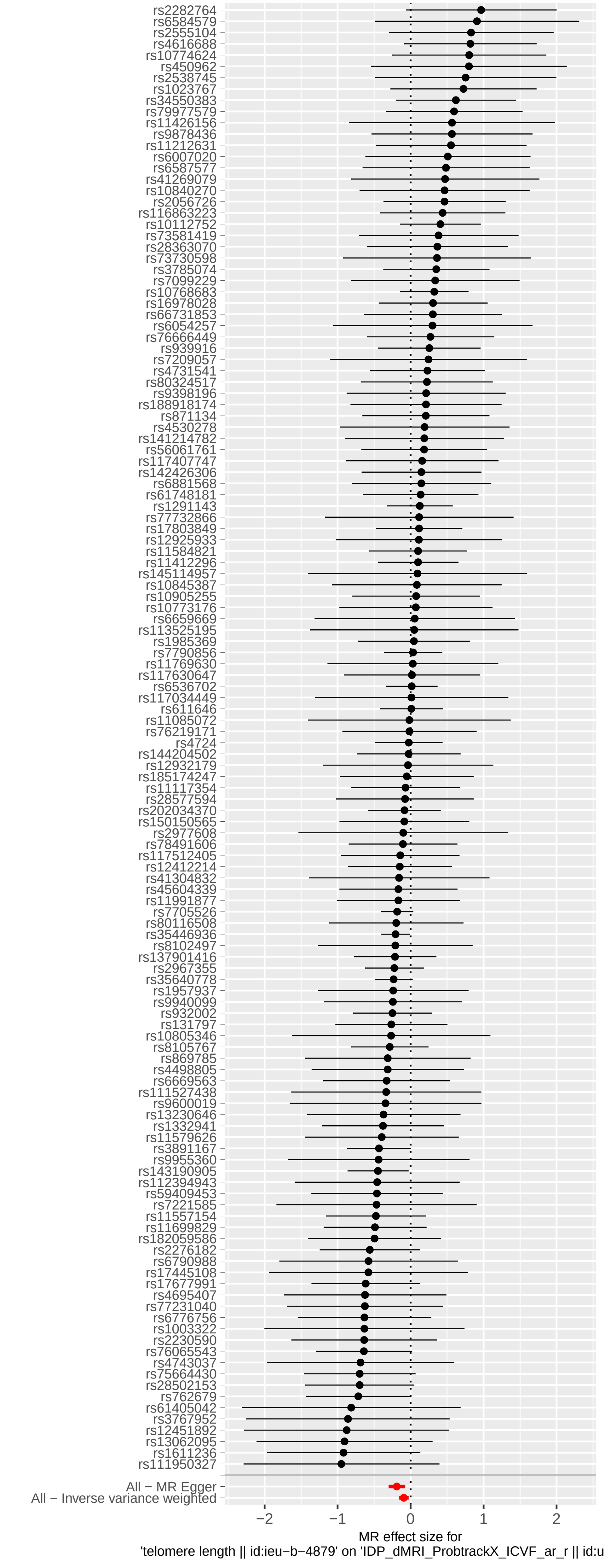
rs2282764
rs6584579
rs2555104
rs4616688
rs10774624
rs450962
rs2538745
rs1023767
rs34550383
rs79977579
rs11426156
rs9878436
rs11212631
rs6007020
rs6587577
rs41269079
rs10840270
rs2056726
rs116863223
rs10112752
rs73581419
rs28363070
rs73730598
rs3785074
rs7099229
rs10768683
rs16978028
rs66731853
rs6054257
rs76666449
rs939916
rs7209057
rs4731541
rs80324517
rs9398196
rs188918174
rs871134
rs4530278
rs141214782
rs56061761
rs117407747
rs142426306
rs6881568
rs61748181
rs1291143
rs77732866
rs17803849
rs12925933
rs11584821
rs11412296
rs145114957
rs10845387
rs10905255
rs10773176
rs6659669
rs113525195
rs1985369
rs7790856
rs11769630
rs117630647
rs6536702
rs117034449
rs611646
rs11085072
rs76219171
rs4724
rs144204502
rs12932179
rs185174247
rs11117354
rs28577594
rs202034370
rs150150565
rs2977608
rs78491606
rs117512405
rs12412214
rs41304832
rs45604339
rs11991877
rs7705526
rs80116508
rs35446936
rs8102497
rs137901416
rs2967355
rs35640778
rs1957937
rs9940099
rs932002
rs131797
rs10805346
rs8105767
rs869785
rs4498805
rs6669563
rs111527438
rs9600019
rs13230646
rs1332941
rs11579626
rs3891167
rs9955360
rs143190905
rs112394943
rs59409453
rs7221585
rs11557154
rs11699829
rs182059586
rs2276182
rs6790988
rs17445108
rs17677991
rs4695407
rs77231040
rs6776756
rs1003322
rs2230590
rs76065543
rs4743037
rs75664430
rs28502153
rs762679
rs61405042
rs3767952
rs12451892
rs13062095
rs1611236
rs111950327
All − MR Egger
All − Inverse variance weighted
−2
−1
0
1
2
MR effect size for
'telomere length || id:ieu-b-4879' on 'IDP_dMRI_ProbtrackX_ICVF_ar_r || id:u
